# Effective Heat and Mass Transport Properties of Anisotropic Porous Ceria for Solar Thermochemical Fuel Generation

**DOI:** 10.3390/ma5010192

**Published:** 2012-01-19

**Authors:** Sophia Haussener, Aldo Steinfeld

**Affiliations:** 1 Institute of Mechanical Engineering, EPFL, Lausanne 1015, Switzerland; 2 Department of Mechanical and Process Engineering, ETH Zurich, Zurich 8092, Switzerland; E-Mail: aldo.steinfeld@ethz.ch; 3 Solar Technology Laboratory, Paul Scherrer Institute, Villigen 5232, Switzerland

**Keywords:** porous media, morphology, transport, radiation, conduction, convection, fluid flow, anisotropy, solar, ceria

## Abstract

High-resolution X-ray computed tomography is employed to obtain the exact 3D geometrical configuration of porous anisotropic ceria applied in solar-driven thermochemical cycles for splitting H${}_{2}$O and CO${}_{2}$. The tomography data are, in turn, used in direct pore-level numerical simulations for determining the morphological and effective heat/mass transport properties of porous ceria, namely: porosity, specific surface area, pore size distribution, extinction coefficient, thermal conductivity, convective heat transfer coefficient, permeability, Dupuit-Forchheimer coefficient, and tortuosity and residence time distributions. Tailored foam designs for enhanced transport properties are examined by means of adjusting morphologies of artificial ceria samples composed of bimodal distributed overlapping transparent spheres in an opaque medium.

## 1. Introduction

H${}_{2}$O/CO${}_{2}$-splitting thermochemical cycles via metal oxide redox reactions are promising thermochemical routes for solar fuel production [1,2]. The 2-step closed material cycle consists of the endothermic reduction of the metal oxide using concentrated solar radiation, followed by the oxidation of the lower-valence oxide with H${}_{2}$O and/or CO${}_{2}$ to produce H${}_{2}$ and/or CO, while regenerating the original metal oxide. Cerium oxide (ceria) has emerged as an attractive candidate for such a cyclic process because it displays rapid fuel production kinetics and high selectivity [3,4,5,6,7,8]. The cycle is described by the pair of redox reactions: 1st step (ceria reduction, 1,800 K)

(1) $${\mathrm{CeO}}_{2-\delta }\to {\mathrm{CeO}}_{2-\delta -x}+x/2O_{2}$$

**Figure 1:**
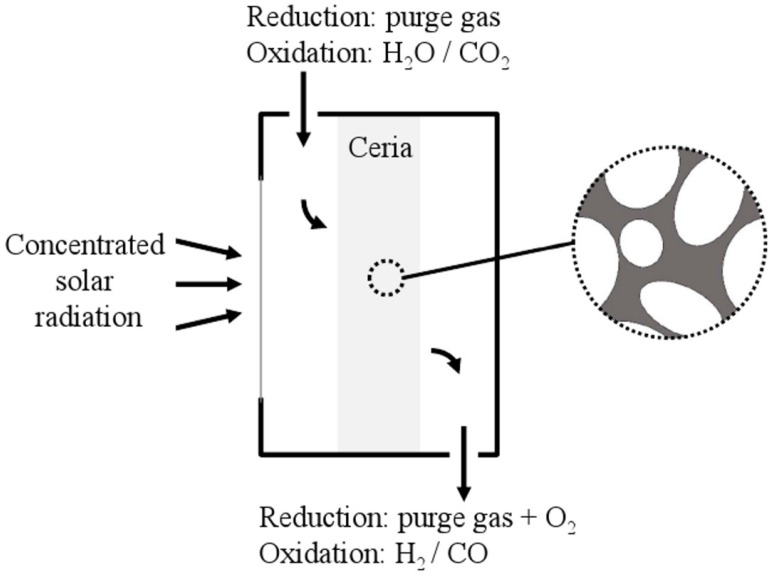
Schematic of the solar reactor for the 2-step solar-driven thermochemical production of fuels. Concentrated solar radiation enters the cavity-receiver through a windowed aperture and is absorbed by the porous ceria (close-up). Reacting gases flow across the porous ceria while product gases exit the cavity.

2nd step (ceria oxidation, 1,100 K)

(2) $${\mathrm{CeO}}_{2-\delta -x}+xH_{2}O\to {\mathrm{CeO}}_{2-\delta }+xH_{2}$$

(3) $${\mathrm{CeO}}_{2-\delta -x}+x{\mathrm{CO}}_{2}\to {\mathrm{CeO}}_{2-\delta }+x\mathrm{CO}$$

where the reduced and oxidized ceria are differentiated by the extent of oxygen nonstoichiometry (δ+x and *δ*, respectively). To ensure efficient heat and mass transport to and from the surface reaction sites and thereby maximize the overall reaction rate, it is found beneficial to utilize the ceria in porous form [3]. When prepared as a porous monolith, the ceria can be incorporated into a solar cavity-receiver and be directly exposed to high-flux solar irradiation while being subjected to the reacting H${}_{2}$O/CO${}_{2}$ flow [9], as schematically shown in Figure 1.

In porous materials, volume-averaging models are commonly applied for heat/mass transfer processes to overcome scale-disparity, *i.e*., the governing equations in the multi-phase media are spatially-averaged and homogenized by the postulation of effective transport properties [10]. However, these properties strongly depend on the inherent structural complexity of the media and, in turn, the averaged models rely heavily on the values of these effective properties. Thus, modeling and optimization of the solar reactor demands for the accurate determination of the effective heat and mass transport properties of the porous ceria. In this study, high-resolution computed tomography (CT) is employed to obtain the 3D digital geometrical representation of the ceria foam, which approaches the exact geometry in the limits of the CT resolution. The CT data are then used in direct pore-level simulations (DPLS) for determining its directional dependent effective transport properties. This approach has been successfully applied to isotropic reticulate porous ceramics [11,12,13,14,15], isotropic packed beds [16,17,18,19,20], and individual transport properties of anisotropic structures [21,22,23,24,25]. The calculated effective heat and mass transport properties can then readily be used in the volume-averaged models [10], accounting for coupled conduction-convection-radiation heat transfer, mass transfer, fluid flow, and chemical reactions. High values for the specific surface area, conductivity, convective heat transfer coefficient, permeability, and tortuosity are targets for the effective transport properties, which should enhance the performance of the solar reactor. In addition, pore-level foam engineering for process optimization is investigated by adjustment of the morphologies and, consequently, the effective transport properties to the specific process needs. This is accomplished through the generation of artificial ceria samples composed of bimodal distributed overlapping transparent spheres in an opaque medium.

## 2. Computed Tomography

**Figure 2:**
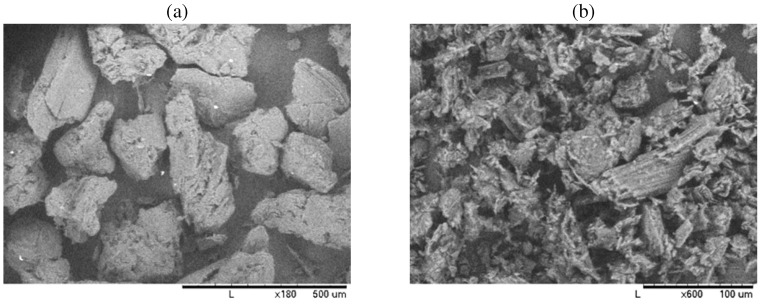

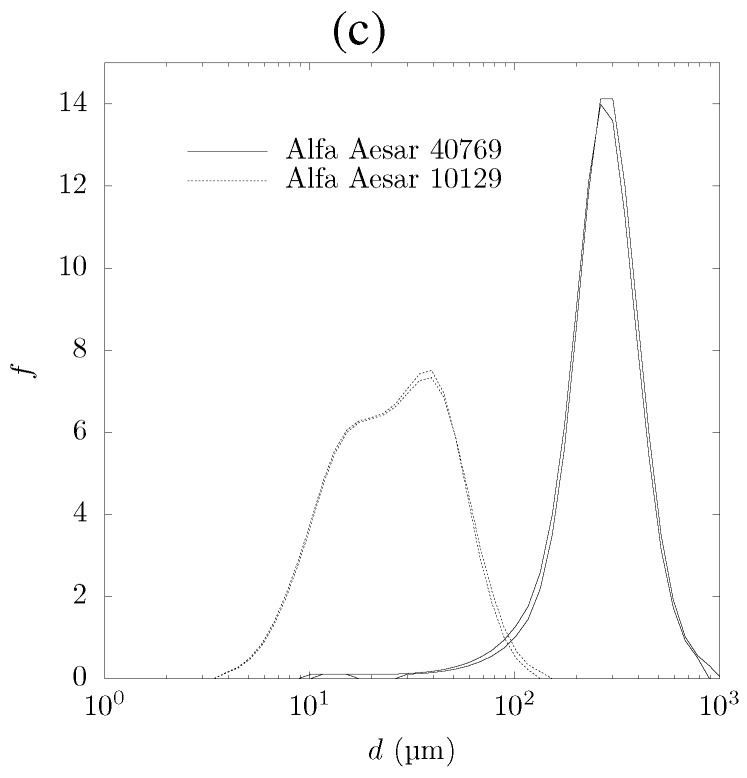
SEM pictures (HORIBA TM1000) of the graphite powder, Alfa Aeasar 40769 (**a**) and Alfa Aesar 10129 (**b**), and volume-based particle-size distributions, *f*, of two random samples of each graphite powder type (**c**).

Three types of porous ceria, each of approximately 65% porosity and prepared using graphite as a sacrificial pore-former, were used for the analysis. Graphite (Alfa Aesar 40769 and 10129, see Figure 2a and b) and ceria (Alfa Aesar 11328) powders are mixed in ethanol for 10 min, dried, uniaxially pressed in a 13 mm-diameter die at 220 MPa for 2 min, and sintered at 1,500 ${}^{\circ }$C for 5 hours (5 and 1 K/min heating and cooling rate, respectively). The three samples differ by their volumetric fractions of the two graphite powder types (Alfa Aesar 10129 and 40769). Laser scattering (HORIBA LA-950) is used for the determination of the particle-size distribution of the sacrificial pore-former, plotted in Figure 2c. Note that these distributions are qualitative as particles are not spherical, which is a base assumption in Mie scattering theory [26] applied for the recalculation of the size distributions. Experimentally determined mean diameters are 28 *μ*m and 270 *μ*m for the Alfa Aesar 10129 and 40769, respectively.

The morphological and effective heat/mass transport properties of the three ceria samples, as determined based on the analysis that follows, are listed in Table 1.

**Table 1:** Volume percent ratio of ceria and graphite particles, numerically and experimentally determined porosity *ε*, numerically determined specific surface $A_{0}$, mean, mode and median diameter *d*, extinction coefficient *β*, fitting parameter *a* of Equation (9), Nu correlation, permeability *K* and Dupuit-Forchheimer coefficient $F_{\mathrm{DF}}$ of the three ceria foam samples in the *x*-, *y*-, *z*-directions.

Sample No.	1	2	3
vol% ratio ^1^	1:3:1	1:2:2	1:1:3
$\varepsilon_{\mathrm{numerical}}$	0.51	0.56	0.55
$\varepsilon_{\mathrm{experimental}}$	0.65 ± 0.1	0.65 ± 0.1	0.65 ± 0.1
$A_{0}$ (mm${}^{-1}$)	672	706	675
$d_{m}$ (*μ*m)	13.7	11.9	10.0
$d_{\mathrm{mode}}$ (*μ*m)	13.3	11.8	8.9
$d_{\mathrm{median}}$ (*μ*m)	13.1	11.5	9.7
*β* (m${}^{-1}$), *x*-direction	30,003 ± 8,282	38,143 ± 6,277	45,173 ± 4,665
*β* (m${}^{-1}$), *y*-direction	31,757 ± 7,067	35,042 ± 4,546	46,277 ± 6,485
*β* (m${}^{-1}$), *z*-direction	69,018 ± 14,735	65,665 ± 9,809	74,835 ± 15,022
*a*, *x*-direction	0.427	0.443	0.506
*a*, *y*-direction	0.354	0.482	0.512
*a*, *z*-direction	0.751	0.740	0.705
Nu, *x*-direction	0.38 + 0.35Re${}^{0.75}$Pr${}^{0.64}$	1.09 + 0.50Re${}^{0.69}$Pr${}^{0.56}$	0.82 + 0.64Re${}^{0.66}$Pr${}^{0.51}$
Nu, *y*-direction	0.37 + 0.41Re${}^{0.68}$Pr${}^{0.58}$	0.75 + 0.53Re${}^{0.68}$Pr${}^{0.59}$	1.13 + 0.56Re${}^{0.69}$Pr${}^{0.51}$
Nu, *z*-direction	1.96 + 0.60Re${}^{0.80}$Pr${}^{0.52}$	1.28 + 0.65Re${}^{0.75}$Pr${}^{0.57}$	1.96 + 0.94Re${}^{0.68}$Pr${}^{0.61}$
*K* (m${}^{2}$), *x*-direction	6.04 × 10${}^{-12}$	3.54 × 10${}^{-12}$	2.92 × 10${}^{-12}$
*K* (m${}^{2}$), *y*-direction	7.97 × 10${}^{-12}$	3.80 × 10${}^{-12}$	3.03 × 10${}^{-12}$
*K* (m${}^{2}$), *z*-direction	7.43 × 10${}^{-13}$	1.27 × 10${}^{-12}$	1.30 × 10${}^{-12}$
$F_{\mathrm{DF}}$ (m${}^{-1}$) , *x*-dir.	18.4 × 10${}^{4}$	16.8 × 10${}^{4}$	19.4 × 10${}^{4}$
$F_{\mathrm{DF}}$ (m${}^{-1}$) , *y*-dir.	12.4 × 10${}^{4}$	17.5 × 10${}^{4}$	19.3 × 10${}^{4}$
$F_{\mathrm{DF}}$ (m${}^{-1}$) , *z*-dir.	278.9 × 10${}^{4}$	93.5 × 10${}^{4}$	75.3 × 10${}^{4}$

^1^ vol% ratio: ceria - Alfa Aesar 40769 - Alfa Aesar 10129.

**Figure 3:**
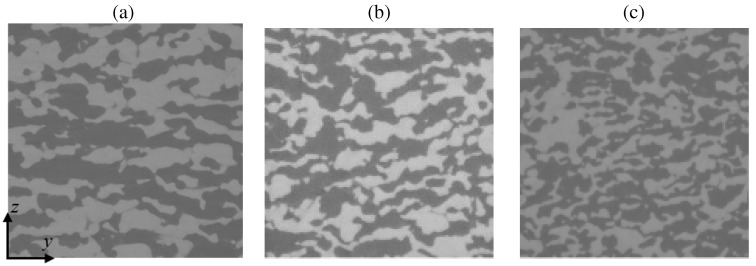
CT scan of the three ceria samples of Table 1: (**a**) No. 1; (**b**) No. 2; and (**c**) No. 3. Edge length of the pictures is 376 *μ*m. Dark is solid phase and bright is void phase.

**Figure 4:**
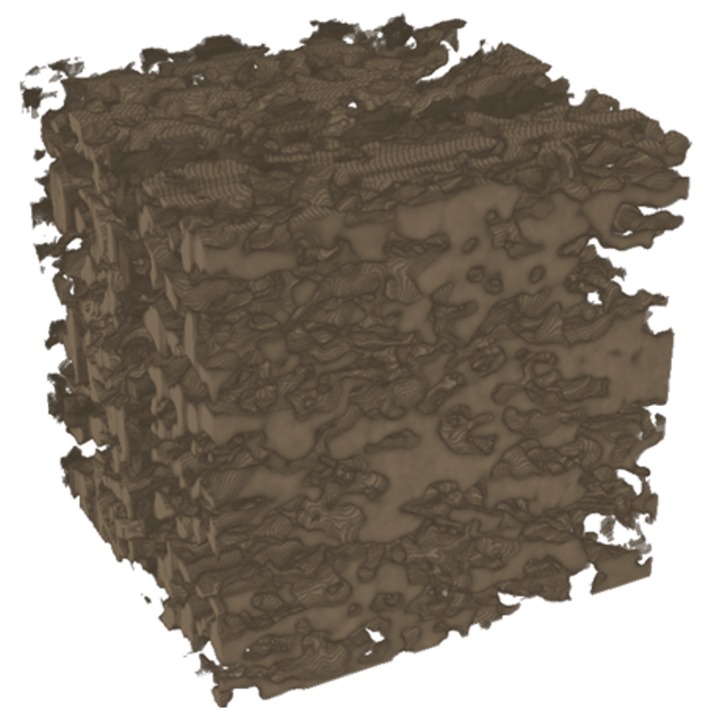
3D rendering of porous ceria sample No. 2. The edge length equals 376 *μ*m.

The inherent morphological anisotropy of the graphite powders and the directional processing creates porous ceria with structural anisotropy. Effective properties of the anisotropic sample are calculated along the three principal directions of the sample: the direction of uniaxial pressing (*z*-direction) and the two orthogonal directions (*x*- and *y*-direction). High-resolution computed tomography of the three samples is obtained with synchrotron radiation of the TOMCAT beamline of the Swiss Light Source (SLS) at the Paul Scherrer Institute (PSI) [27,28] with the following operating conditions: 36 keV photon energy, 400 *μ*A beam current, 100 *μ*m-Al/10 *μ*m-Fe/40 *μ*m-Cu filter, 2.1 s exposure time, and 1500 projections. The datas voxel size is 0.37 *μ*m and the field of view (FOV) investigated is 0.76 × 0.76 × 0.76 mm${}^{3}$. A CT scan of each sample is shown in Figure 3. Exemplary, a 3D rendered picture of sample No. 2 is shown in Figure 4. The discrete absorption values obtained by CT are linearly interpolated in 3D to obtain a continuous representation of the phase boundary. The tomographic data is segmented via the mode method.

## 3. Morphological Characterization

**Figure 5:**
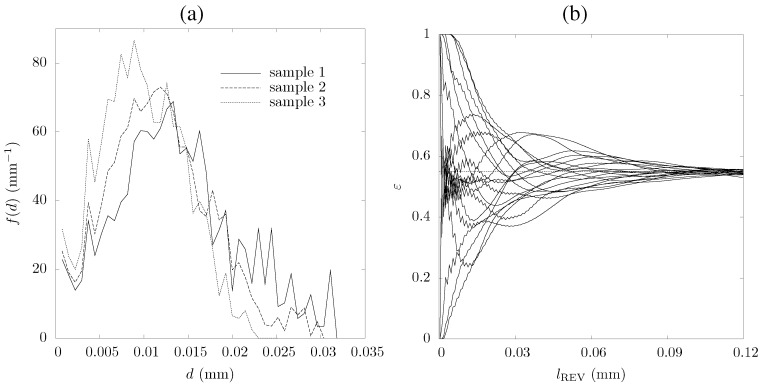
Pore-size distribution of the three ceria samples (**a**), and porosity calculated for sample No. 3 in 20 subsequently growing volumes (**b**). The dotted horizontal line indicates $\varepsilon_{\mathrm{numerical}}$ to which the values converge.

The tomography-based methodology [12] is applied to determine the foam’s porosity *ε*, specific surface $A_{0}$, and pore size distribution f(d). Monte Carlo sampling is used to compute the two point correlation function,

(4) $$s_{2}\left(r\right)=\frac{\int_{V}\int_{4\pi }\Psi \left(x\right)\Psi \left(x+r\hat{s}\right)d\hat{s}dx}{V4\pi }\;$$

for which $s_{2}\left(0\right)=\varepsilon$, and $\left(ds_{2}/dr\right){|}_{r=0}=-A_{0}/4$. Ψ(r) is the pore-scale indicator function, which equals 1 if r lies in the void phase and 0 if r lies in the solid phase. Alternatively, porosity can be calculated by a voxel-based approach leading to the same results for the samples at hand. An opening operation (dilation and subsequent erosion) with a spherical structuring element of diameter *d* is used to determine.

(5) $$f\left(d\right)=-\frac{d\varepsilon_{\mathrm{op}}\left(d\right)}{\varepsilon dd}\;$$

where $\varepsilon_{\mathrm{op}}$ is the porosity calculated after an opening [29]. The representative elementary volume (REV), the smallest volume for continuum domain, is determined by calculating the porosity for sub-volumes with increasing volume size until the variation in porosity is negligible, e.g., varying within a band of ε±γ [11,12]. The mean intercept length is calculated to get a quantification of the samples’ anisotropy. Calculated sample porosities and specific surface areas are given in Table 1. The underestimation of the calculated porosity compared to the experimentally determined one (obtained by weight measurements) is attributed to the limited resolution of the tomography data. Correspondingly, the specific surface area calculated is expected to be underestimated since the resolution of surface irregularities is limited by the voxel size of the CT data. The calculated pore-size distributions, shown in Figure 5a, correspond to the lower size limit because of the spherical structuring element used for the opening operation. Squeezing, shrinking, and break-up of the pores during the ceria foam processing (pressing and sintering) leads to smaller pore sizes than expected by the size distribution of the pore-former graphite particles. Sample No. 3 is prepared by the largest volumetric fraction of small graphite particles (Alfa Aesar 10129) and therefore has a slightly larger fraction of small pores. Calculated mean, mode, and median diameter of the particle size distributions are given in Table 1. The edge length, $l_{\mathrm{REV}}$, of a cubic representative elementary volume is determined based on porosity calculations on subsequently growing volumes. The calculated porosity as a function of $l_{\mathrm{REV}}$ for 20 randomly chosen growing volumes in sample No. 3 is shown in Figure 5b. $l_{\mathrm{REV}}$ for the three samples investigated are 0.11 mm, 0.10 mm, and 0.06 mm, respectively, assuming a porosity band of ±0.06 to be sufficient. For the following calculations, the minimum sample size is given by REV.

**Figure 6:**
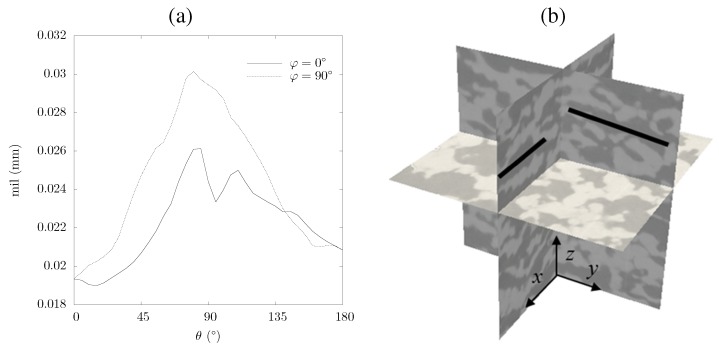
Mean intercept length for ceria sample No. 1 as a function of *θ* at *φ* = 0 and 90${}^{\circ }$ (a), and three orthogonal planes of sample No. 1 with black bars indicating the direction of the elongated pores (b).

The mean intercept length for sample No. 1 is shown in Figure 6a. Mean intercept length is defined as the average distance between two solid-fluid boundaries [30]. *θ* and *φ* represent the polar and azimuthal angles in spherical coordinates. Scattering in the data is due to statistical variation in the morphology. The intercept length along the *x*- and *y*-axis (*θ* = 90${}^{\circ }$ and *φ* = 0${}^{\circ }$, and *θ* = 90${}^{\circ }$ and *φ* = 90${}^{\circ }$, respectively) is 25 to 35% larger because, due to uniaxial pressing, the pores in *x*- and *y*-directions are elongated and channel-like structures develop, while the pores in *z*-direction are squeezed. This is exemplary shown in Figure 6b by the three orthogonal planes of sample No. 1 and the two black bars indicating elongated pores and channels evolving along the *x*- and *y*-axis.

## 4. Heat Transfer Characterization

### 4.1. Radiation Heat Transfer

The solid volume of the ceria foam is assumed opaque, while the void volume is assumed to be transparent ($\sigma_{s,d}=\kappa_{d}=0$), as the gas absorption coefficients of the reacting gases are orders of magnitude smaller than the absorption of radiation at the solid-fluid boundary. For example, the Planck mean absorption coefficient of CO at 1000 K and 1 atm is 2.1 m${}^{-1}$, calculated based on the HITRAN2004 database [31]. The volume-averaged radiative transfer equation (RTE) is given by [32].

(6) $$\hat{s}\cdot \nabla I_{i}\left(x,\hat{s}\right)=-\beta I_{i}\left(x,\hat{s}\right)+\kappa_{d}I_{b,i}\left(x,\hat{s}\right)+\frac{\sigma_{s}}{4\pi }\int_{\Omega_{i}=0}^{4\pi }L_{i}\left(x,{\hat{s}}_{i}\right)\;\Phi \left({\hat{s}}_{i},\hat{s}\right)d\Omega_{i}$$

Collision-based Monte Carlo method is applied to compute the cumulative distribution functions of the radiation attenuation path and of the cosine of incidence at the solid wall, from which *β* and Φ are extracted [20]. The albedo, $\sigma_{s}/\beta$, is solely dependent on the surface reflectivity of the phase boundary. A sample of 0.37 × 0.37 × 0.37 mm${}^{3}$, corresponding to 1000 × 1000 × 1000 voxels, is investigated. The extinction coefficients along the *x*-, *y*-, *z*-directions are listed in Table 1. These values are comparable with those obtained using a correlation for reticulate porous ceramics ($\beta =\frac{4.4(1-\varepsilon )}{d}$) [33]. The calculated extinction coefficients show the same order of magnitude as those estimated by transmittance measurements of similar ceria foams (ε=0.72, graphite and starch pore-formers) at low wavelengths where the opacity assumption of solid ceria holds [34]. The porous ceria foam behaves as a nearly opaque medium, as expected for small pore dimensions. Due to uniaxial pressing, the pores in *x*- and *y*-directions are elongated, while the pores in *z*-direction are squeezed and, consequently, lead to shorter extinction path lengths and larger extinction coefficients. In addition, the increasing fraction of smaller pores from sample No. 1 to 3 leads to increased attenuation. This trend is less pronounced in the *z*-direction because circumferentially squeezing of an oblate by a factor of two leads to its elongation by a factor of 4. Note that the uniaxial pressing is not identical for the three samples, resulting in different grades of anisotropy.

### 4.2. Conduction Heat Transfer

The temperature distribution and the corresponding heat flux within a porous sample in a 1D domain is directly linked to the effective thermal conductivity by [10,11,14,35]:

(7) $$k_{e}=l\frac{-\int_{A_{s}}k_{s}\nabla T_{s}\cdot \hat{n}dA_{s}-\int_{A_{f}}k_{f}\nabla T_{f}\cdot \hat{n}dA_{f}}{\left(T_{1}-T_{2}\right)\left(A_{s}+A_{f}\right)}\;$$

**Figure 7:**
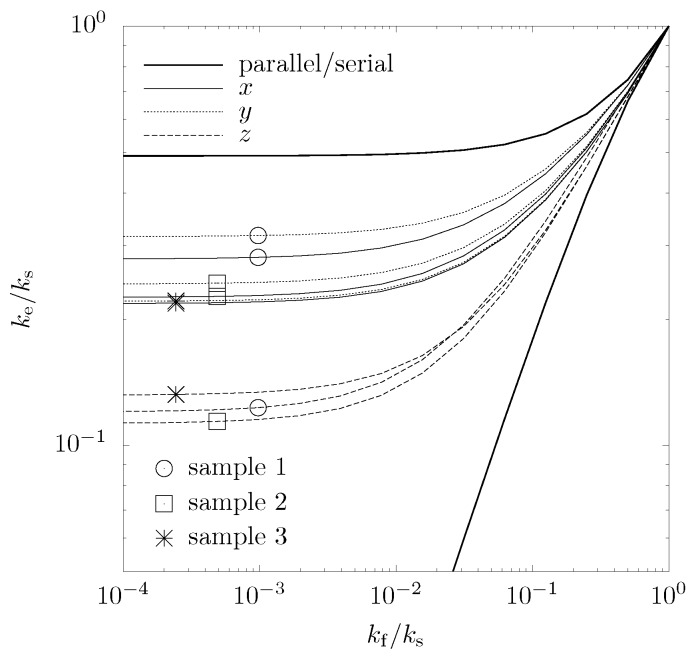
Effective normalized conductivity of the three samples (Table 1) as function of the ratio fluid-to-solid conductivities in the three directions.

This requires the solution of the pore-level steady-state heat conduction equations within the solid and the fluid phases for a cubic sample,

(8) $$\nabla \cdot \left(k_{s}\nabla T_{s}\right)=0\;$$

(9) $$\nabla \cdot \left(k_{f}\nabla T_{f}\right)=0\;$$

with boundary conditions at solid-fluid interface: $T_{s}=T_{f}$, $\hat{n}\cdot k_{s}\nabla T_{s}=\hat{n}\cdot k_{f}\nabla T_{f}$, at the lateral walls: $q\cdot \hat{n}=0$, at inlet: $T_{s}=T_{f}=T_{1}$, and at outlet: $T_{s}=T_{f}=T_{2}$. The finite volume (FV) technique with successive over-relaxation is applied to solve Equations 8–9. A sample of 0.37 × 0.37 × 0.37 mm${}^{3}$, corresponding to 1,000 × 1,000 × 1,000 voxels, is investigated. A mesh composed of cubic finite volumes is used for the calculations. Grid convergence is obtained with mesh element size of 21.5 *μ*m. The calculated effective thermal conductivity normalized by the solid conductivity is shown in Figure 7 as a function of the ratio fluid-to-solid conductivities, $k_{f}/k_{s}$. Also included in the graph are the maximum and minimum conductivities possible in a regular ordered two-phase media, described by serial and parallel slab models [35]. As expected, the conductivity of the foam lies within these boundaries.

As the fluid conductivity decreases, the influence of the sample morphology becomes more pronounced while conduction in the fluid is less important. Due to the uniaxial pressing, the structures in the *x*- and *y*-directions are aligned more parallel to the heat flux and exhibit enhanced conductivities compared to that in the *z*-direction. Again, samples No. 2 and No. 3 show less pronounced anisotropy compared to sample No. 1. An analytical description of the effective thermal conductivity as a function of $k_{f}/k_{s}$ is derived by fitting the numerically determined curves to a weighted sum of parallel and serial slab models,

(10) $$\frac{k_{e}}{k_{s}}=a\frac{\frac{k_{f}}{k_{s}}}{\varepsilon \left(1-\frac{k_{f}}{k_{s}}\right)+\frac{k_{f}}{k_{s}}}+\left(1-a\right)\left(\varepsilon \frac{k_{f}}{k_{s}}+1-\varepsilon \right)$$

The fitted parameter a is given in Table 1 for three samples and in the three directions. The RMS of the fitting is less than 0.165.

### 4.3. Convection Heat Transfer

**Figure 8:**
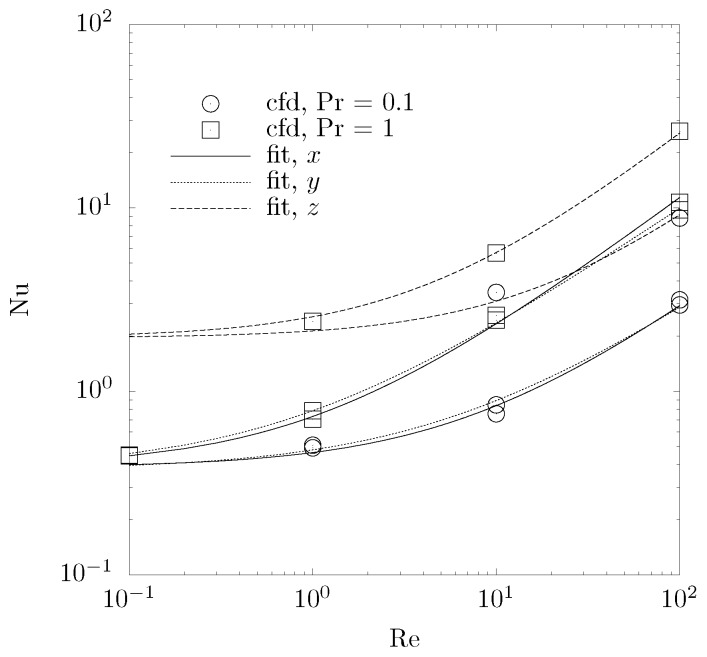
Nu number as a function of Re and Pr numbers (points) and fit (lines) for sample No. 1 in the three directions.

The coupled continuity, momentum, and energy equations are solved in the fluid phase at the pore-level using a CFD code [36] within a square duct containing a sample of the ceria foam to obtain the temperature distribution in the fluid phase and the heat flux through the solid-fluid interface [11,15]. The effective heat transfer coefficient is defined as:

(11) $$h_{\mathrm{sf}}=\frac{\int_{A_{\mathrm{sf}}}q^{\prime \prime }dA_{\mathrm{sf}}}{A_{\mathrm{sf}}\Delta T_{\mathrm{lm}}}\;$$

where $A_{\mathrm{sf}}$ is the solid-fluid boundary, $q^{\prime \prime }$, the heat flux through the boundary, and $T_{\mathrm{lm}}$ the logarithmic mean temperature. A sample of 0.37 × 0.37 × 0.19mm^3^, corresponding to × 1000 × 1000 × 500 voxels, is investigated. The smallest dimension lies in the main flow direction. Convergence of the numerical calculations is achieved for a terminal residual RMS of the iterative solution below 10^−5^ and for a maximal mesh element length of 3 μm. The mesh is generated with an in-house mesh generator for unstructured body-fitted grids, which covers the domain by tetrahedral elements and subsequently refines the elements at the phase boundary by rounding, cutting, and smoothing [37]. The resulting grid has between 50 × 10^6^ and 150 × 10^6^ mesh elements. The calculated Nu as a function of Re for Pr = 0.1 and 1, is shown in Figure 8 in the three directions. Re = u_D_*d*_m_*ρ/μ* and Pr = *c_p_μ/k*_f_, where *d*_m_ denotes the calculated mean pore diameter and u_D_ the superficial velocity. The curves are fitted to a correlation of the form:

(12) $${\text{N}}_{\text{u}}=a_{1}+a_{2}{\text{Re}}^{a3}{\text{Pr}}^{a4}$$

with the constans *a*_1_ to *a*_4_ given in Table 1. The Rms of the fitting is less than 0.6. The heat transfer coefficient increases in the *z*-direction because of the more tortuous path for fluid flow, increasing the accessible surface area for solid-fluid heat exchange. This trend is most pronounced for sample No. 1. Values for Nu lie above those experimentally measured for ceramic foams [38], but within the range of those for packed beds [39].

## 5. Mass Transfer Characterization

**Figure 9:**
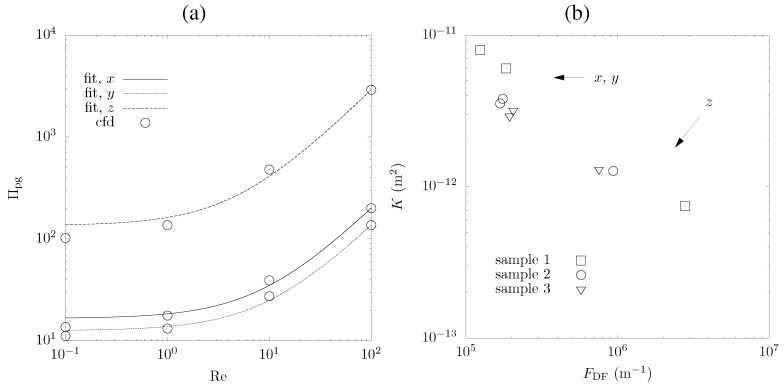
(**a**) Normalized pressure drop as a function of Re number for the three directions in sample No. 1; (**b**) Calculated permeability *vs*. Dupuit-Forchheimer coefficient for the three samples and in the three directions.

Fluid flow across porous media can be described by the extended Darcy’s law [33],

(13) $$0=-\nabla p-\frac{\mu }{K}u_{D}-\rho F_{\mathrm{DF}}\left|u_{D}\right|u_{D}\;$$

which relates the normalized pressure gradient, $\Pi_{\mathrm{pg}}=\frac{\nabla pd^{2}}{\mu u_{D}}$, to the Re number. CFD at the pore level in the laminar fluid phase is applied to determine the permeability, *K*, and Dupuit-Forchheimer coefficient, $F_{\mathrm{DP}}$, [11,15], as well as the tortuosity and residence time distributions [11,40]. $F_{\mathrm{DF}}$ accounts for the inertia-induced resistance term, which comes into play at high Re. Sample size, mesh element size, and convergence criterion are identical as for the convection calculations. $\Pi_{\mathrm{pg}}$, for sample No. 1 is shown in Figure 9.a as a function of Re for the three directions. The Dupuit-Forchheimer term comes into play at Re > 0.5. Calculated *K* and $F_{\mathrm{DP}}$ are given in Table 1 and Figure 9.b for the three directions. Channels in the *x*- and *y*-directions that evolved during the uniaxial pressing lead to smaller pressure gradients and, consequently, higher permeabilities. The Dupuit-Forchheimer term decreases as well, since smaller inertia forces apply to fluid particles flowing through the foam in the *x*- and *y*-directions in a less disturbed manner. This trend is again most pronounced for sample No. 1. Comparison of the calculated *K* with estimates by the capillary model ($K=2.03\times 10^{-12}$ m${}^{2}$) [35], hydraulic radius model ($K=1.25\times 10^{-12}$ m${}^{2}$) [35], and fibrous bed model ($K=4.73\times 10^{-13}$ m${}^{2}$) [41], and of the calculated $F_{\mathrm{DP}}$ with estimates by extended Ergun equation ($F_{\mathrm{DP}}=22.9\;\times \;10^{4}$ m${}^{-}1$) [42], for *ε* = 0.65 and *d* = 10 *μ*m, show reasonable agreement, considering the differences in morphology.

**Figure 10:**
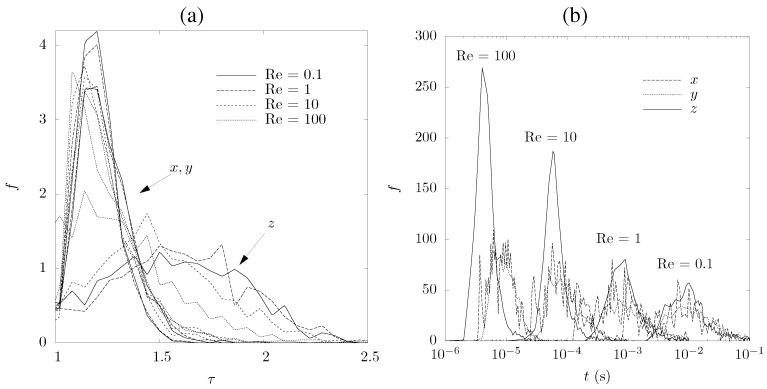
Tortuosity (**a**) and residence time (**b**) distributions for Re = 0.1 to 100 for sample No. 1, sample length of 0.17 mm, and for the three directions.

Tortuosity is defined as the ratio of the real length of the connected pore channels to the thickness of the porous sample in the main flow direction. Residence time is defined as the time required for a fluid particle to flow through the porous sample. Tortuosity and residence time distributions (for a sample length of 0.17 mm) are calculated based on a large number of stream lines, which are determined with the aid of the pore-level velocity distributions previously calculated. The results for sample No. 1 are shown in Figure 10. Tortuosities in the *x*- and *y*-directions are smaller and show narrower peaks than that in the *z*-direction, as the fluid is able to pass through the foam along the channels formed during the uniaxial pressing. Tortuosity along the *z*-direction decreases as Re increases because of the evolution of vortices in partially connected pores, blocking them and forcing the fluid to flow through more direct, undisturbed paths. In general, the larger the tortuosity the larger the accessible specific surface area for the heterogeneous thermochemical reaction. Residence time distributions in the *z*-direction show a sharper peak than that in the two other directions, especially at large Re. At low Re, the distribution show a tail due to fluid particles temporarily trapped in partially connected pores or death ends. Calculated mean tortuosities and residence times are given in Table 2 for Re = 1.

**Table 2:** Mean, mode, and median tortuosity and residence time for sample No. 1 in the three directions for a sample with length 0.17mm at Re = 1.

Direction	$\tau_{m}$	$\tau_{\mathrm{mode}}$	$\tau_{\mathrm{median}}$	$t_{m}$ (s)	$t_{\mathrm{mode}}$ (s)	$t_{\mathrm{median}}$ (s)
*x*	1.24	1.20	1.22	0.0026	0.0006	0.0018
*y*	1.20	1.20	1.19	0.0028	0.0007	0.0020
*z*	1.61	1.80	1.59	0.0018	0.0009	0.0011

For the determination of the dispersion tensor, D, the previously calculated velocity vector field is used. The solution of the transient species conservation equation with convection and diffusion links the axial (subscript a) and radial (subscript r) part of D to the Gaussian distributed concentration at a specific time by [43]:

(14) $$S_{i}=\sqrt{2D_{i}t}\;$$

where $S_{i}$ represents the standard deviation of the Gaussian distribution. The calculated D for sample No. 1 is given by:

(15) Dr,x/y=8.32×10-5Re0.558

(16) Da,x/y=1.19×10-3Re0.487

for the *x*- and *y*-direction, while for the *z*-direction it is given by

(17) Dr,z=9.56×10-5Re0.562

(18) Da,z=2.00×10-3Re0.514

## 6. Tailored Foam Design

**Figure 11:**
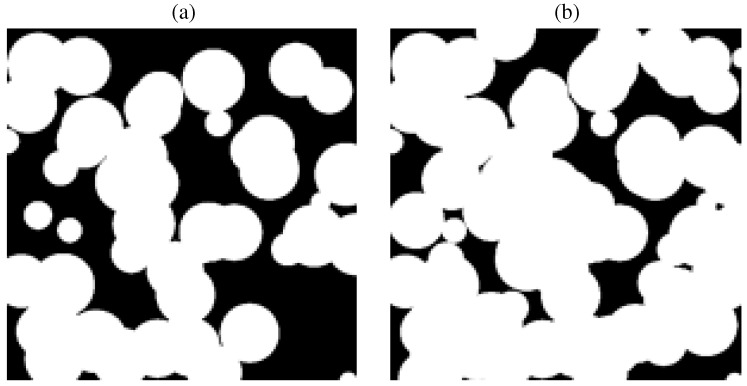
2D slice through the artificial BDOTS samples with *ξ* = 1, and $\varepsilon_{\mathrm{BDOTS}}$ = 0.6 (**a**) and 0.8 (**b**). Edge length is 540 *μ*m.

Optimization of the thermochemical process and the associated solar reactor is closely related to enhanced heat and mass transport within the porous material. Our pore-level engineering approach in this regard is based on artificially generated morphologies that are input to the previously described DPLS methodology. Morphologies composed of bimodal distributed overlapping transparent sphere (BDOTS) in an opaque ceria matrix are examined, as these allow for a structured investigation of the morphology-property relation and for an adjustment of the effective transport properties to the specific process needs. BDOTS are employed because they closely resemble the ceria foams fabricated by the sacrificial pore-former process with two different pore-former graphite particle types (Alfa Aesar 10129 and 40769) with two distinct mean particle sizes (see Figure 2c). The porosity of BDOTS is calculated by

(19) $$\varepsilon_{\mathrm{BDOTS}}=1-\exp\left(-\frac{4\pi }{3}n\left[\xi r_{1}^{3}+\left(1-\xi \right)r_{2}^{3}\right]\right)\;,$$

where *n* represents the number density of carbon particles, *ξ* represents the number fraction of graphite particles or pores with 100 *μ*m diameter ($r_{1}$ = 50 *μ*m), and 1-ξ the number fraction of graphite particles or pores with 10 *μ*m diameter ($r_{2}$ = 5 *μ*m). A 2D slice through the samples is shown in Figure 11. Exemplary, Nu, *K*, and $F_{\mathrm{DP}}$ are calculated for samples made of ξ=1 and $\varepsilon_{\mathrm{BDOTS}}=0.6$ and 0.8.

**Figure 12:**
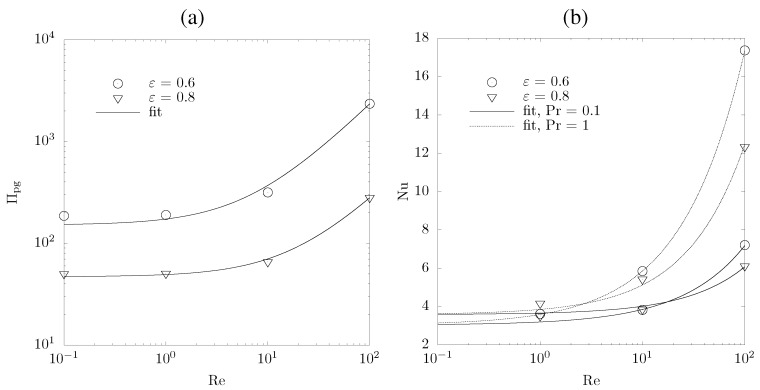
(**a**) Normalized pressure gradient as a function of Re; and (**b**) Nu numbers as function of Re for Pr = 0.1, and 1, for the artificially generated porous sample (*ξ* = 1, and $\varepsilon_{\mathrm{BDOTS}}$ = 0.6 and 0.8).

Figure 12a shows the normalized pressure gradient over the porous sample. The resulting *K* and $F_{\mathrm{DP}}$ are given in Table 3. *K* increases considerably (by more than an order of magnitude) when the sample porosity is increased, consistent with the hydraulic radius model and fibrous bed model [35,41]. Note that the capillary model, which only linearly relates *K* and *ε*, underestimates this dependence. In contrast, $F_{\mathrm{DP}}$ decreases with increasing porosity as a result of less tortuous paths for fluid flow across the sample, reducing the inertia induced forces. This is consistent with the model of $F_{\mathrm{DP}}$ being proportional to $\left(1-\varepsilon \right)/\varepsilon^{3}$ [42]. For low Re numbers (Re < 20 for Pr = 0.1, and Re < 3 for Pr = 1), heat transfer is favored for samples with high porosity, see Figure 12b. The opposite is true for high Re numbers because vortices evolve in partially open pores (cavities) and obstruct the passage of fluid flow. Comparison of the three ceria samples to the results of the BDOTS at $\varepsilon_{\mathrm{BDOTS}}$ = 0.6 shows that Nu numbers are considerably lower in the real samples. This is expected as $r_{1}$ (= 50 *μ*m) is larger than the calculated mean diameters of the ceria samples (see Table 1). Correspondingly, *K* is larger for the BDOTS.

**Table 3:** Permeability, Dupuit-Forchheimer coefficient, and Nu correlation for the artificial porous sample with *ξ* = 1, and $\varepsilon_{\mathrm{BDOTS}}$ = 0.6 and 0.8.

$\varepsilon_{\mathrm{BDOTS}}$	*K* (m${}^{2}$)	$F_{\mathrm{DF}}$ (m${}^{-1}$)	Nu
0.6	6.62 × 10${}^{-11}$	219143	3.03 + 0.55Re${}^{0.71}$Pr${}^{0.54}$
0.8	2.13 × 10${}^{-10}$	23315	3.59 + 0.28Re${}^{0.76}$Pr${}^{0.55}$

The complete variation of the $\varepsilon_{\mathrm{BDOTS}}-r_{1}-r_{2}-\xi -$set and the calculation of the corresponding effective transport properties enable in-depth understanding of the morphology-property relations and, consequently, guide the pore-level engineering.

## 7. Summary and Conclusions

Ceramic foams of ceria, used in redox reactions for the solar thermochemical splitting of H${}_{2}$O and CO${}_{2}$, were analyzed for their morphological characteristics and their effective transport properties. The samples were produced by a sacrificial pore-former process, resulting in two main pore sizes with structural anisotropy. Their 3D micro-geometries were obtained by high-resolution tomography and were incorporated in direct pore-level numerical simulations to determine anisotropic effective heat transfer properties (extinction coefficients, thermal conductivities, and heat transfer coefficients) and mass transfer properties (permeabilities, Dupuit-Forchheimer coefficients, dispersion tensors, tortuosity and residence time distributions). Within the limits of the numerical truncation error (*i.e*., mesh refinement) and the accuracy of geometrical representation (*i.e*., statistical variations and CT resolution), the DPLS applied on the CT-based 3D geometry approaches the exact solution. Due to uniaxial pressing (*z*-direction), channels evolved along the *x*- and *y*-directions, resulting in increased radiative extinction along the *z*-direction. The thermal conductivity along the *x*- and *y*-directions was enhanced due to the more parallel alignment of the structure to the heat flux in these directions. Convective heat exchange along the *z*-direction was enhanced due to the larger tortuosity. Calculated Nu values were within those predicted for packed beds. Permeability was higher while the Dupuit-Frochheimer coefficient was lower in the *z*-direction compared to those in the other directions, and showed reasonable agreement with those calculated by capillary, hydraulic radius, fibrous bed and extended Ergun equation models. Artificial porous ceria samples composed of bimodal distributed overlapping transparent spheres in an opaque medium—resembling the morphology of the ceria foam—were examined for enhanced transport properties. Permeability increased while Dupuit-Forchheimer coefficient decreased with increasing porosity. For large Re numbers, lower porosity resulted in enhanced convective heat transfer, while the opposite was true for high Re numbers because vortices evolved in partially open pores and obstructed the passage of fluid flow. The morphology-property relations and guidelines for pore-level engineering were elucidated.

The calculated effective properties can be applied in volume-averaged governing equations for modeling and optimization of the solar reactor configuration.
